# A Mini-Review of In Vitro Data for *Candida* Species, Including *C. auris,* Isolated during Clinical Trials of Three New Antifungals: Fosmanogepix, Ibrexafungerp, and Rezafungin

**DOI:** 10.3390/jof10050362

**Published:** 2024-05-20

**Authors:** Ana Espinel-Ingroff, Nathan P. Wiederhold

**Affiliations:** 1 Virginia Commonwealth University Medical Center, Richmond, VA 23219, USA; 2 Department of Pathology and Laboratory Medicine, University of Texas Health Science Center at San Antonio, San Antonio, TX 78229, USA; wiederholdn@uthscsa.edu

**Keywords:** fosmanogepix, ibrexafungerp, rezafungin, *Candida* species, clinical trials, in vitro activity

## Abstract

This mini-review summarizes the clinical outcomes and antifungal susceptibility results, where available, for three new antifungals, including fosmanogepix, ibrexafungerp, and rezafungin, against *Candida* isolates cultured from patients in clinical trials. When reported, most of the data were generated by the Clinical and Laboratory Standards Institute (CLSI) broth microdilution method or by both the CLSI and European Committee on Antimicrobial Susceptibility Testing (EUCAST) methodologies. For fosmanogepix, we summarize the in vitro data for *C. auris* isolates from 9 patients and for *Candida* spp. cultured from 20 patients in two clinical trials. Ibrexafungerp has also been evaluated in several clinical trials. From conference proceedings, a total of 176 *Candida* isolates were evaluated in the FURI and CARES studies, including 18 *C. auris* isolates (CARES study). However, MIC data are not available for all clinical isolates. Results from the ReSTORE rezafungin phase 3 clinical study also included in vitro results against *Candida* spp., but no patients with *C. auris* infections were included. In conclusion, this mini-review summarizes insights regarding clinical outcomes and the in vitro activity of three new antifungals against *Candida* spp. cultured from patients in clinical trials.

## 1. Introduction

The incidence of fungal infections is increasing worldwide and is associated with significant morbidity and mortality in immunocompromised patients [1,2]. There are several antifungals with novel mechanisms of action that have recently been approved for clinical use in patients or are in the last stage of clinical development. These include ibrexafungerp and rezafungin, which have been approved by the U.S. Food and Drug Administration (FDA) and are available for clinical use, and fosmanogepix, which has completed phase 2 clinical studies and for which a phase 3 study is soon to begin [3,4,5,6]. The development of new antifungal agents is important because the incidence of fungal infections and rates of drug resistance both continue to increase [7,8]. These new agents may play a vital role against infections caused by *Candida* spp. with reduced susceptibility or resistance to clinically available drugs, such as *C. glabrata* and *C. auris*. This is especially important against *C. auris*, an emerging pathogen, that continues to spread throughout the world and was associated with a dramatic peak in the U.S. in 2021 [8]. Unfortunately, screening is not conducted uniformly in the U.S., so the true burden of *C. auris* cases is most likely underestimated. In addition, pan-resistant *C. auris* infections have been documented [9], and fatal cases of breakthrough candidiasis caused by this pathogen have been reported in individuals receiving combination therapy liposomal amphotericin B and caspofungin [10]. The echinocandin resistance in these breakthrough strains was demonstrated to be due to a novel mutation in the hot-spot 1 region of the *FKS1* gene.

## 2. Purpose Statement

In this mini-review, we focused on the clinical and in vitro data from patients with invasive disease caused by *Candida* spp. cultured from patients enrolled in the clinical trials of three new antifungal agents: fosmanogepix, ibrexafungerp, and rezafungin.

## 3. Fosmanogepix

**Figure 1:**
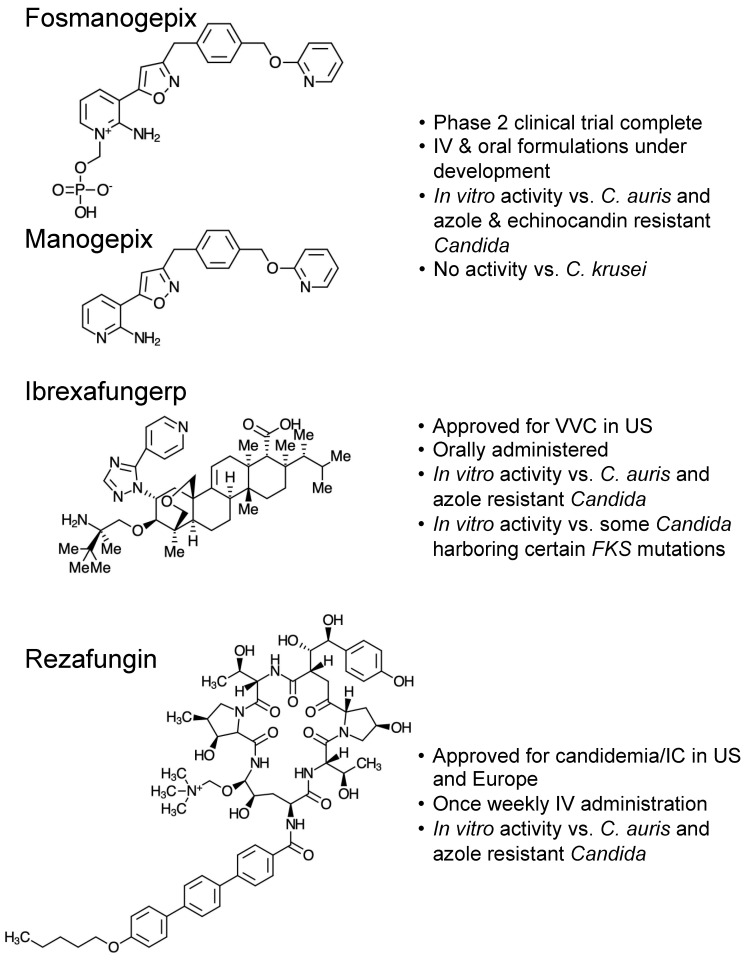
Structures and characteristics of fosmanogepix/manogepix, ibrexafungerp, and rezafungin.

Fosmanogepix (FMGX, PF-07842805, APX001, E1211) is a prodrug that is converted in vivo to the active moiety manogepix (MGX, APX001A, E1210) by systemic phosphatases following administration (Figure 1) [6]. Manogepix has a novel mechanism of action inhibiting the fungal acyltransferase enzyme Gwt1, which is an important component of the glycosylphosphatidylinositol (GPI)-anchored protein maturation pathway [11,12]. This agent has broad-spectrum activity against many pathogenic yeast, molds, and dimorphic fungi (e.g., *Coccidioides* spp.), including *C. auris* and azole-resistant *Aspergillus fumigatus* [6,13]. However, it has limited activity against certain *Candida* spp., including *C. krusei* and the rarer species *C. inconspicua* and *C. kefyr* [14]. The in vitro potency of manogepix has also translated into in vivo efficacy in murine models of invasive candidiasis caused by *C. albicans*, *C. glabrata*, and *C. auris* [15,16,17]. Pharmacokinetic/pharmacodynamic (PK/PD) studies have demonstrated concentration-dependent activity with AUC/MIC being the parameter most closely associated with efficacy [15,16]. Due to its potent in vitro activity, in vivo efficacy in experimental models of *Candida* infections, considerable bioavailability following oral administration in humans, extensive tissue penetration, and a favorable safety profile, fosmanogepix may become a treatment option in patients with invasive candidiasis, including those with chorioretinitis or meningitis [6,14,17,18,19,20]. Both IV and oral formulations received fast-track and orphan drug designations from the U.S. FDA for invasive mycoses caused by *Candida, Aspergillus, Cryptococcus, Coccidoides, Scedosporium, Fusarium* spp., and the Mucorales (*Mucor* and *Rhizopus* spp.), and invasive candidiasis by the European Medicines Agency (EMA).

## 4. Phase 2 Clinical Trials of Fosmanogepix in the Treatment of Invasive Candidiasis/Candidemia, including Infections Caused by *Candida auris*

The results of two phase 2 clinical trials that evaluated fosmanogepix in the treatment of patients with candidiasis have been published. One study conducted in intensive care units in South Africa was an open-label, multicenter, single-arm study that evaluated patients 18 years of age or older who had invasive candidiasis or candidemia caused by *C. auris* [21]. A total of 9 patients were included and received fosmanogepix 1000 mg IV twice daily on day 1, followed by 600 mg daily thereafter with the option to switch to oral therapy at 800 mg once daily from days 4 through 42. Treatment was deemed to be successful in 8 of 9 (89%) patients (survival and clearance of *C. auris* from blood/tissue cultures without the need for additional antifungals at the end of the study treatment). Two patients experienced serious adverse events, and two patients died during the study. Both the adverse effects and the deaths were considered unrelated to fosmanogepix therapy. Both CLSI and EUCAST MIC results for manogepix and other antifungals against *C. auris* isolates collected at baseline are shown in Table 1. These in vitro results are similar to what others have reported for manogepix against larger sets of isolates [18,22].

A second phase 2 study evaluated the efficacy of fosmanogepix for first-line treatment of non-neutropenic patients with candidemia [23]. Patients with infections caused by *C. krusei* were excluded. Fosmanogepix was administered IV for 14 days, beginning with a loading dose of 1000 mg twice daily on day 1 and then 600 mg daily thereafter. Patients could be switched to oral therapy (700 mg once daily) after day 4. Success, defined as clearance of *Candida* from blood cultures and survival to day 42, was observed in 80% (16 of 20 patients) in the modified intent-to-treat population, and the mean time to the first negative blood culture was 2.4 days. Manogepix MICs against the 22 *Candida* baseline isolates cultured from the 20 patients are shown in Table 2. These in vitro results are also similar to what others have reported for manogepix against common *Candida* spp. [14,24,25]. One patient experienced recurrent candidemia due to *C. glabrata* two weeks after the end of treatment, and the manogepix MIC increased from 0.004 mg/L at baseline to 0.12 mg/L (>30-fold increase). In contrast, in another patient who experienced a clinical relapse secondary to a *C. albicans* biliary infection, the manogepix MIC remained the same (0.008 mg/L) between the baseline bloodstream and subsequent biliary isolates.

## 5. Ibrexafungerp

Ibrexafungerp (SCY-078 or MK-3118, Brexafemme) is a semi-synthetic derivative of enfumafungin, a naturally occurring product, and is the first member of the triterpenoid class of antifungal agents [26]. Although both the echinocandins and triterpenoids target the production of 1,3-β-D-glucan in the cell wall of pathogenic fungi through non-competitive inhibition of the 1,3-β-D-glucan synthase complex, ibrexafungerp is structurally different than the echinocandins, and the binding sites only partially overlap [27,28,29]. This results in limited cross-resistance between these different antifungal classes. However, certain mutations can affect both the echinocandins and ibrexafungerp, including the F659del and F659S mutations in hot-spot 1 of Fks1p [30,31,32,33,34]. In addition, it is now known that mutations both upstream (i.e., E655A) and downstream (W715L) of this hot-spot region can also markedly reduce the in vitro activity of ibrexafungerp. Furthermore, this agent can be given orally, while the other echinocandins must be administered intravenously. In preclinical animal models, ibrexafungerp has demonstrated in vivo activity against *C. albicans*, *C. parapsilosis*, and echinocandin-susceptible and resistant strains of *C. glabrata* [35,36], as well as against *C. auris*, both in disseminated and cutaneous models of infection [36,37], which is consistent with the in vitro activity reported by different groups [31,38,39,40,41,42]. In one of the largest preclinical in vitro studies of ibrexafungerp against *C. auris*, the MIC of ibrexafungerp ranged from 0.06 to 2 mg/L with a mode of 1 mg/mL [42]. Concentration-dependent activity has also been observed with ibrexafungerp in animal models of candidiasis, and AUC/MIC is the PK/PD parameter most closely associated with in vivo efficacy [35,43]. These in vitro and in vivo results are promising and point to the potential use of this agent for controlling skin infection and colonization of patients, as well as in the treatment of invasive diseases.

## 6. Phase 2 and 3 Clinical Trials of Ibrexafungerp in the Treatment of Invasive Candidiasis/Candidemia, Including Infections Caused by *Candida auris*

Although both oral and IV formulations have been evaluated in preclinical studies, the clinical data are for the oral formulation and in patients refractory to or intolerant of standard antifungal therapy [4]. These have included several studies of patients with vulvovaginal candidiasis (VVC), for which ibrexafungerp is approved for clinical use in the U.S. [44,45], as well as in patients with invasive candidiasis [46,47,48,49,50]. In one phase 2 study that evaluated oral ibrexafungerp as step-down therapy following initial IV echinocandin treatment, favorable responses were observed in 6 of the 7 that received ibrexafungerp at a maintenance dose of 750 mg per day following an initial loading dose of 1250 mg [51]. Population pharmacokinetic assessment predicted that this dose would achieve target exposure in ~85% of patients established in a preclinical study of invasive candidiasis [43]. Similar responses were observed in patients randomized to a lower dose of ibrexafungerp (1000 mg loading dose followed by 500 mg per day) or standard of care (fluconazole 800 mg oral loading dose followed by 400 mg daily thereafter). Ibrexafungerp was well tolerated at both dose levels.

Two phase 3 open-label, single-arm studies (FURI, NCT03059992, for the treatment of patients intolerant to or refractory to standard care, and CARES, NCT03363841, for the treatment of *C. auris* infections) have also evaluated the safety and efficacy of oral ibrexafungerp, although results from these studies are only available in abstract form thus far. These studies have included patients with candidemia and multiple forms of invasive candidiasis, including intraabdominal, bone/joint, oropharyngeal, esophageal, and vulvovaginal candidiasis, among others, and infections caused by azole and echinocandin-resistant strains. In one update from the FURI and CARES studies, results from patients with candidemia or invasive candidiasis were reported [52]. Complete or partial responses were observed in 13 of 18 patients with candidiasis and 20 of 30 with invasive candidiasis. Of the major pathogens cultured from patients in this study (*C. albicans* and *C. glabrata*), response rates (clinical improvement, complete or partial response) were 11 of 16 for *C. albicans* and 7 of 7 for *C. glabrata* [53]. In the CARES study, 14 of 18 patients infected with *C. auris* demonstrated complete or partial response with ibrexafungerp treatment [54]. Antifungal susceptibility was also determined in the FURI study for cultures collected at screening and all subsequent study visits. This included 158 *Candida* spp. [48], and MIC results for ibrexafungerp and fluconazole are shown in Table 3.

## 7. Rezafungin

Rezafungin (CD101, Rezzayo) is a new echinocandin that has been approved for the treatment of candidemia and invasive candidiasis in the U.S. and Europe. As with the other echinocandins, rezafungin non-competitively inhibits the 1,3-β-D-glucan synthase enzyme complex leading to reductions in 1,3-β-D-glucan levels in the cell wall of many pathogenic fungi [55]. Similar to the other members of this class, it must also be administered intravenously. However, due to a modification of its structure which stabilizes the molecule, the half-life of rezafungin is ~130 h, which allows for once-weekly administration, which is in contrast to the other echinocandins that must be administered daily. Based on its slow clearance, rezafungin is administered as a loading dose of 400 mg followed by weekly doses of 200 mg thereafter. This strategy also takes advantage of the pharmacokinetic/pharmacodynamic parameters of AUC/MIC and Cmax/MIC that have been associated with in vivo efficacy for the echinocandins [56,57,58,59,60,61]. The in vitro activity of rezafungin is similar to that of the other echinocandins [62,63,64,65], and preclinical in vivo models have reported effectiveness against infections caused by different *Candida* spp., including *C. albicans*, *C. auris*, *C. dubliniensis*, *C. glabrata*, *C. parapsilosis*, and *C. tropicalis* [15,59,60,66,67,68]. As with the other echinocandins, point mutations in highly conserved regions of the *FKS1* and *FKS2* genes can lead to resistance to rezafungin [63,69].

## 8. Phase 2 and 3 Clinical Trials of Rezafungin in the Treatment of Invasive Candidiasis/Candidemia

Rezafungin has been evaluated for the treatment of candidemia and invasive candidiasis in two randomized clinical trials. In the STRIVE study (NCT02734862), a phase 2 double-blind trial, patients were randomized to receive two different doses of rezafungin (400 mg weekly or 400 mg loading dose followed by 200 mg weekly) or the standard dose of caspofungin [70]. *Candida albicans* was the predominant pathogen (49.7%), followed by *C. glabrata* (20.2%), *C. parapsilosis* (15.3%), *C. tropicalis* (12.0%), *C. krusei* (2.7%), *C. dubliniensis* (2.7%), and other *Candida* spp. (4.4%). Overall cure, defined as resolution of signs of candidemia/invasive candidiasis and mycological eradication, was reported in 60.5% (46/76) of those in the rezafungin 400 mg weekly group, 76.1% (35/46) in the rezafungin 400 mg loading dose/200 mg weekly group, and 67.2% (41/61) in the caspofungin group. Day 30 all-cause mortality rates were 15.8%, 4.4%, and 13.1%, respectively. In each group, the antifungal agents were well tolerated, and the drug-related serious adverse events were between 1.2% to 2.9% of the patients.

ReSTORE was a randomized, double-blind, multicenter phase 3 study (NCT03667690) in which patients with candidemia or invasive candidiasis were treated with either rezafungin (400 mg loading dose followed by 200 mg weekly) or caspofungin [71]. Two primary endpoints were evaluated: global cure (clinical/radiological cure and mycological eradication) at day 14, and all-cause mortality at day 30. Results between the two groups were similar, with 55 of 93 (59%) patients in the rezafungin group and 57 of 94 (50%) achieving global cure by day 14. All-cause mortality was also similar between the groups with 24% in the rezafungin group and 21% in the caspofungin group dying or having an unknown survival status at day 30. The distribution of *Candida* species in this study was similar to that in the STRIVE study, with >99% being susceptible to both caspofungin and rezafungin using CLSI breakpoints. Neither the STRIVE nor ReSTORE trials included patients infected with *C. auris.* In a pooled analysis of the modified intent-to-treat population from both the STRIVE and ReSTORE trials, the 30 all-cause mortality rates were the same between the rezafungin 400 mg/200 mg and caspofungin groups (19%) [72]. Mycological eradiation by day 5 occurred in 73% of those treated with rezafungin and 65% of those with caspofungin. An analysis of ReSTORE clinical outcomes data by baseline *Candida* species and in vitro susceptibility results has recently been published (Table 4) [73]. Only two patients in this study had baseline isolates that had non-susceptible MIC results to both rezafungin and caspofungin. Both were randomized to the rezafungin group and were classified as treatment successes based on the day 30 all-cause mortality endpoint.

## 9. In Vitro Guidance

Currently, no breakpoints have been set for manogepix or ibrexafungerp to classify individual isolates of different *Candida* spp. as susceptible or resistant to these antifungals. In addition, epidemiologic cut-off values (ECVs or ECOFFs) have not been formally determined for either of these agents. Instead, individual laboratories have reported what is called wild-type upper-limit (WT-UL) MIC values to help distinguish between wild-type (isolates unlikely to have acquired resistance) from non-wild type (those that may harbor resistance mechanisms) for both manogepix and ibrexafunerp (Table 5) [14,18,24,34,38,39,74,75,76,77]. In contrast, susceptible breakpoints have now been set for rezafungin by the U.S. FDA and CLSI [78,79]. It should be noted that no breakpoints for rezafungin resistance are currently set, and the FDA and CLSI breakpoints differ against some *Candida* species.

## 10. Conclusions

Each of these new antifungal agents, fosmanogepix, ibrexafungerp, and rezafungin, has demonstrated promising in vitro and in vivo activity against *Candida* spp., including *C. auris*. In addition, positive outcomes have also been reported in phase 2 and phase 3 clinical trials, although available data are limited to small phase 2 studies thus far for fosmanogepix, and results are only available in abstract form for ibrexafungerp. To date, the most robust clinical data and in vitro data from clinical trials are available for rezafungin, although no patients with *C. auris* were enrolled, and only two patients had infections at baseline that were considered non-susceptible to this echinocandin. Thus, while these results are promising, more studies are needed to determine the role of these antifungal agents in the treatment of infections caused by *Candida* isolates that are resistant to the azoles and/or the echinocandins.

## Figures and Tables

**Table 1:** CLSI and EUCAST MIC ranges for manogepix and other antifungal agents against 9 baseline *Candida auris* isolates cultured from patients enrolled in a phase 2 clinical trial. All results are in mg/L.

Antifungal Agent	CLSI MIC Range	EUCAST MIC Range
Manogepix	0.008–0.015	0.004–0.015
Amphotericin B	1	0.5
Anidulafungin	0.5–1	0.03–2
Micafungin	0.12–0.25	0.06–2
Fluconazole	≥128	≥128
Voriconazole	1–2	0.5–8

**Table 2:** CLSI MIC ranges for manogepix and other antifungal agents against 22 baseline *Candida* species isolates from a phase 2 clinical trial of patients with candidemia. All results in mg/L.

Species (No.)	Manogepix	Anidulafungin	Fluconazole	Amphotericin B
*C. albicans* (8)	0.002–0.008	0.016–0.03	0.12–2	0.5–4
*C. dubliniensis* (1)	0.004	0.016	0.12	1
*C. glabrata* (10)	0.004–0.03	0.03–0.25	0.5–4	0.5–2
*C. parapsilosis* (3)	0.004–0.016	0.5–4	0.25–0.5	0.5–4

**Table 3:** Ibrexafungerp and fluconazole susceptibility results against 158 *Candida* isolates from the FURI study. All results were determined by CLSI broth microdilution methods and are reported in mg/L.

Species (No.)	MIC Parameter	Ibrexafungerp	Fluconazole
*C. glabrata* (92)	Range	0.12–8	≤0.06–>64
	MIC_50_	0.5	2
	MIC_90_	4	32
*C. albicans* (45)	Range	0.03–8	≤0.06–>64
	MIC_50_	0.06	4
	MIC90	0.5	>64
*C. krusei* (5)	Range	0.5–1	2–16
*C. parapsilosis* (5)	Range	0.12–0.25	0.25–2
*C. tropicalis* (4)	Range	0.06–0.5	0.5–64

**Table 4:** Outcomes in relation to *Candida* spp. and rezafungin MIC values from the ReSTORE phase 3 study. MICs were determined by the CLSI broth microdilution methodology.

Species	Outcome	Number of Patients with Response/Number by Pathogen and MIC Value (mg/L)								
		0.008	0.015	0.03	0.06	0.12	0.25	0.5	1	2
*C. albicans*	Cure	4/7	11/20	4/6	1/4	1/2	---	---	---	---
	Eradication	5/7	11/20	5/6	1/4	1/2	---	---	---	---
*C. glabrata*	Cure	---	---	6/8	4/6	6/9	---	0/1	---	---
	Eradication	---	---	7/8	6/6	6/9	---	1/1	---	---
*C. tropicalis*	Cure	---	3/3	5/8	5/7	1/2	---	---	---	---
	Eradication	---	3/3	5/8	5/7	2/2	---	---	---	---
*C. parapsilosis*	Cure	---	---	---	---	---	---	1/1	2/4	3/3
	Eradication	---	---	---	---	---	---	1/1	2/4	3/3

**Table 5:** Wild-type upper-limit (WT-UL) MICs for manogepix and ibrexafungerp, and susceptible breakpoints for rezafungin against different *Candida* species. All values in mg/L.

Wild-Type Upper-Limit MIC Values for Manogepix and Ibrexafungerp				
Species	Manogepix		Ibrexafungerp	
	EUCAST	CLSI	EUCAST	CLSI
*C. albicans*	0.06	0.015–0.03	0.25–0.5	0.5
*C. auris*	0.06	0.03	2	---
*C. glabrata*	0.12	0.12–0.25	0.5–1	2
*C. krusei*	---	---	1–4	4
*C. parapsilosis*	0.06	0.03	1–4	1
*C. tropicalis*	0.016	0.06	1–2	1
**Rezafungin Breakpoints against *Candida* spp.**				
**Species**	**U.S. FDA Breakpoint**		**CLSI Breakpoint**	
*C. albicans*	≤0.12		≤0.25	
*C. auris*	---		≤0.5	
*C. dubliniensis*	---		≤0.12	
*C. glabrata*	≤0.12		≤0.5	
*C. krusei*	---		≤0.25	
*C. parapsilosis*	≤2		≤2	
*C. tropicalis*	≤0.12		≤0.25	

## Data Availability

No new data were created or analyzed in this study. Data sharing is not applicable to this article.
